# Detection of vascularity in wrist tenosynovitis: power doppler ultrasound compared with contrast-enhanced grey-scale ultrasound

**DOI:** 10.1186/ar3185

**Published:** 2010-11-09

**Authors:** Andrea S Klauser, Magdalena Franz, Rohit Arora, Gudrun M Feuchtner, Johann Gruber, Michael Schirmer, Werner R Jaschke, Markus F Gabl

**Affiliations:** 1 Department of Radiology, Medical University Innsbruck, Anichstr. 35, Innsbruck, 6020, Austria; 2 Department of Internal Medicine, Medical University Innsbruck, Anichstr. 35, Innsbruck, 6020, Austria; 3 Department of Trauma Surgery, Medical University Innsbruck, Anichstr. 35, Innsbruck, 6020, Austria

## Abstract

**Introduction:**

We sought to assess vascularity in wrist tenosynovitis by using power Doppler ultrasound (PDUS) and to compare detection of intra- and peritendinous vascularity with that of contrast-enhanced grey-scale ultrasound (CEUS).

**Methods:**

Twenty-six tendons of 24 patients (nine men, 15 women; mean age ± SD, 54.4 ± 11.8 years) with a clinical diagnosis of tenosynovitis were examined with B-mode ultrasonography, PDUS, and CEUS by using a second-generation contrast agent, SonoVue (Bracco Diagnostics, Milan, Italy) and a low-mechanical-index ultrasound technique. Thickness of synovitis, extent of vascularized pannus, intensity of peritendinous vascularisation, and detection of intratendinous vessels was incorporated in a 3-score grading system (grade 0 to 2). Interobserver variability was calculated.

**Results:**

With CEUS, a significantly greater extent of vascularity could be detected than by using PDUS (*P *< 0.001). In terms of peri- and intratendinous vessels, CEUS was significantly more sensitive in the detection of vascularization compared with PDUS (*P *< 0.001). No significant correlation between synovial thickening and extent of vascularity could be found (*P *= 0.089 to 0.097). Interobserver reliability was calculated to be excellent when evaluating the grading score (κ = 0.811 to 1.00).

**Conclusions:**

CEUS is a promising tool to detect tendon vascularity with higher sensitivity than PDUS by improved detection of intra- and peritendinous vascularity.

## Introduction

Besides mechanical overloading and attrition, rheumatologic diseases are widespread causes of tenosynovitis and tendinosis. These chronic systemic inflammatory diseases lead to enormous costs for hospitalizations, physician visits, employee's illness, and invalidity pensions. They are caused not only by osseous destruction, but also by tendinosis and consecutive tendon rupture, which are not detectable by conventional imaging such as radiographs. Rheumatoid arthritis (RA), with a prevalence of 0.5% to 1%, the most common disease of this group [1], is accompanied by tendon involvement in approximately 40% [2]. Flexor digitorum, extensor digitorum, and extensor carpi ulnaris tendons are frequently involved in early RA [3-5]. Tenosynovitis of extensor carpi ulnaris can be its first manifestation [4].

Angiogenesis is a hallmark of acute inflammation and exacerbation of chronic disease. Neovascularization in the synovial membrane is considered to be an important process in early pathogenesis as well as in the perpetuation and progression of RA [6,7]. Disordered angiogenesis promotes the proliferation and invasion of the tenosynovium [8]. Finally, tenosynovial invasion is associated with an increased tendon-rupture rate and a poor prognosis for long-term hand function [8-10]. Besides, angiogenesis is a step in the inflammatory cascade that can be identified and quantified with imaging modalities [5].

Despite the great involvement of tendons in RA, little research has been done into imaging of tendon disease. Color and power Doppler ultrasound (CDUS/PDUS) have been shown to be of diagnostic value in the detection of vascularity in synovial proliferation [11,12]. Doppler US, however, is limited in the detection of slow flow and flow in the small vessels of angiogenesis present in synovial proliferations [13].

Newer contrast-specific US modes based on the higher harmonic emission capabilities of second-generation contrast agents allow imaging with grey-scale US and the use of a lower, nondestructive US power (very low mechanical index, MI = 0.06 to 0.1). This avoids Doppler-specific artefacts like blooming and aliasing and permits continuous imaging without the need for time intervals between scans for contrast replenishment [14]. Contrast-enhanced grey-scale ultrasound (CEUS) compared with PDUS has already been shown to improve significantly the detection of vascularity in joints of patients with RA [15]. Furthermore, Song *et al*. [16] reported on a higher sensitivity of CEUS in the detection of vascularity in comparison with contrast-enhanced (CE) MRI in examining patients with knee osteoarthritis [16]. To our knowledge, only one study has been published using CEUS to detect vascularity in healthy tendons [17].

The goal of this study was to assess the value of PDUS and CEUS in the detection of tendon hypervascularity and to evaluate a reliable quantification for tendon involvement in rheumatic diseases.

## Materials and methods

From March 2004 to January 2006, 26 tendons in 24 patients (nine men, 15 women; mean age ± SD: 54.4 ± 11.8 years) underwent B-mode, PDUS, and CEUS examination. Retrospective evaluation of 14 extensor and 12 flexor tendons of the wrist was carried out for this study by including two different tendons in two patients examined at different appointments with a time interval of at least 6 months for the two patients.

Written informed consent according to the Declaration of Helsinki was obtained by all patients, and approval by our university ethics committee was obtained. The patients were recruited consecutively, according to their referral from the rheumatology outpatient clinic and Traumatology Department.

Clinical activity was evaluated by considering the presence of reddening, swelling, pain, or a combination of these. Subsequently, US scanning of the clinically active or suggestive tendon was performed by one examiner.

Of the 24 patients, 19 (79.2%) previously were diagnosed with rheumatic diseases [16 (66.7%) with RA and one (4.2%) each with morbus Still, scleroderma, and spondyloarthropathy]. These diagnoses are based on the 1987 revised criteria of the American College of Rheumatology [18], on the European Spondyloarthropathy Study Group criteria [19], and modified New York criteria [20], respectively. The remaining five (20.8%) patients showed tendinosis from overuse.

Blood tests were performed to determine serologic activity, including erythrocyte sedimentation rate (ESR; with the Westergren method) and rheumatoid factors (RFs; with enzyme-linked immunosorbent assay for IgM-RF). Fourteen (73.7%) of the ESR tests resulted in increased values (mean ESR, 30.9 mm/h). RFs were positive in 11 of the sera (mean value, 498.6 kU/L; range, 22 to 2,920 kU/L). Finally, nine patients were tested positive for anticyclic citrullinated peptide antibodies (anti-CCP).

### Ultrasound techniques

We used an MPX-Technos unit fitted with high-frequency transducers (LA424, LA LA532, Esaote, Genoa, Italy) for the US examinations.

### Grey-scale ultrasound and power Doppler ultrasound

Grey-scale US was performed according to a standardized protocol by using 13.0 MHz and the musculoskeletal program presets, which remained fixed throughout the examination. PDUS was performed with standardized machine settings by using a frequency of 10.0 to 12.5 MHz with a pulse repetition frequency of 750 to 1,000 kHz, a low wall filter, and medium persistence. The window (colour box) was restricted to the vascular area studied. After visualization of colour-flow signals, pulsed wave spectral Doppler imaging was performed using the lowest filter setting and the smallest scale available that would display the Doppler waveforms as large as possible without aliasing. A spectral Doppler tracing was obtained to confirm that the PDUS signals represented true arterial or venous flow.

Grey-scale US and PDUS were performed for adequate delineation of the tendon and to assess the presence of peritendinous effusion and tenosynovial thickening.

Subsequently, PDUS was performed to detect tenosynovitis, which was defined as hypoechoic or anechoic thickened tissue, which is seen in two perpendicular planes and which may exhibit Doppler signal, according to the Outcome Measures in Rheumatology Clinical Trials (OMERACT) criteria [21]. If vascularity was found with PDUS, the presence of active tenosynovitis was determined. Lack of vascularity confirmed the diagnosis of effusion or inactive tenosynovitis.

### CEUS

The agent was prepared in a standard manner with a dosage of 4.8 ml SonoVue flushed with 10 ml saline. Subsequently, US scanning by using a low-MI (*≤ *0.1) technique, CnTI (Contrast tuned Imaging; Esaote, Genoa, Italy), was performed to ensure sufficient enhancement after bolus administration, allowing an examination window of up to 5 minutes.

**Figure 1:**
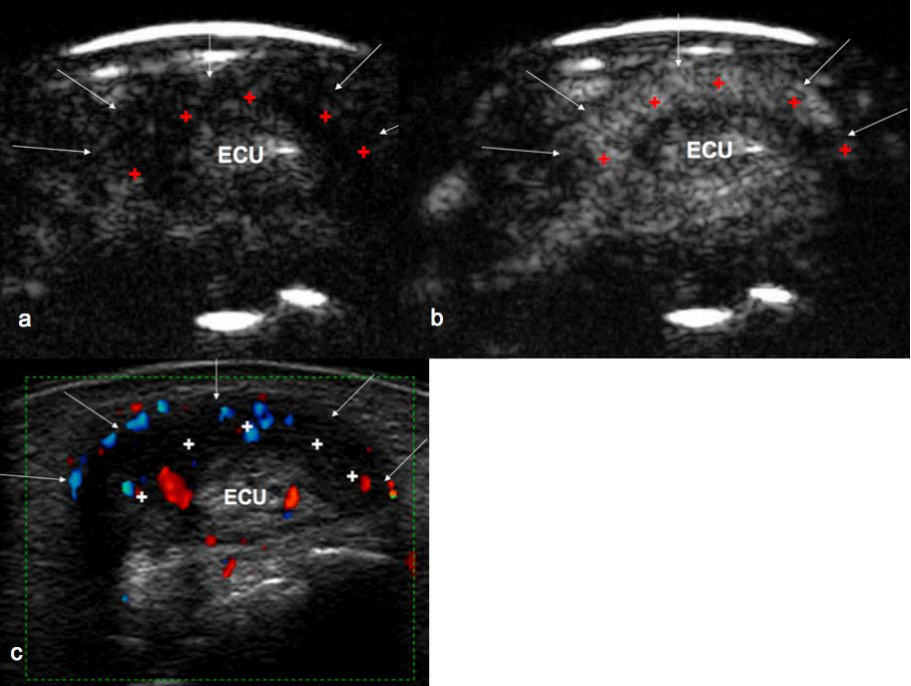
**Transverse plane at the wrist through extensor carpi ulnaris tendon**. **(a) **CEUS examination with hypoechoic peritendinous space before contrast medium washin. **(b) **Hyperechoic peritendinous space and intratendinous enhancement after contrast medium washin. **(c) **PDUS examination. Grade 2 in every scoring system. Arrows, border of tendon sheath; cross, synovial thickening; ECU, extensor carpi ulnaris tendon.

CEUS was used to assess the amount of inactive and active tenosynovitis. Modified accordingly the OMERACT criteria [21], active tenosynovitis was defined as thickening of the synovium within the tendon sheath that exhibits contrast enhancement in two perpendicular planes (see Figure 1).

Examinations were carried out by a single radiologist, experienced in musculoskeletal US for 7 years.

Images and clips were analyzed after digital storage on the hard disc by two examiners.

### Subjective grading

**Figure 2:**
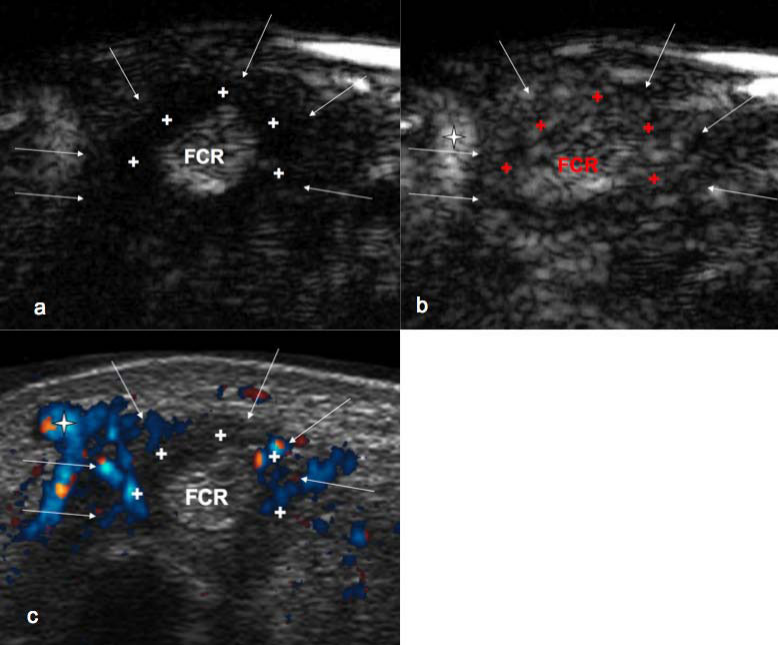
**Transverse plane at the wrist through flexor carpi radialis tendon**. **(a) **CEUS examination with hypoechoic peritendinous space before contrast medium washin. **(b) **Hyperechoic peritendinous space, tendon after contrast medium washin (grade 2). **(c) **With PDUS, intratendinous vessels are not displayed (grade 1). Arrows, Border of tendon sheath; cross, synovial thickening; star, radial artery; FCR, flexor carpi radialis tendon.

Inflammation was graded subjectively by using a 3-point grading scale (see Table 1) according to following criteria: 1, extent of synovial proliferation (synovial thickness) measured in the axial plane in mm; 2, extent of the vascularized pannus detected with PDUS and CEUS, respectively, in relation to the extent of the whole synovial proliferation; In detail, the extent of vascularization referred to the amount of synovial proliferation (already determined by thickness measurement) exhibiting vascularity in the axial scanning plane. Extent of vascularisation was graded as grade 1 when more than 50% avascular synovial proliferation could be seen than in active synovitis, and as grade 2 when more than 50% of synovitis appeared to be vascularized. 3, detection of intratendinous or solely peritendinous vessels, located in the tendon sheath; and 4, intensity of peritendinous enhancement in comparison with extratendinous enhancement, which was assessed outside the tendon sheath (see Figure 2).

**Table 1:** Subjective grading of vascularity in tenosynovitis

	Synovial thickness (grey-scale US)	Extent of vascularity (PDUS, CEUS)	Peri- and intratendinous vessel detection (PDUS, CEUS)	Intensity of peri- to extratendinous vascularity (CEUS)
Grade 0	<2 mm	No vascularity	No vascularity	No vascularity
Grade 1	2 to 4 mm	Extent <50%^a^	Solely peritendinous	Peri- <extratendinous
Grade 2	>4 mm	Extent ≥50%^a^	Peri- and intratendinous	Peri ≥ extratendinous

^a^50% of the peritendinous synovial proliferation in the axial scanning plane. CDUS, color Doppler ultrasound; PDUS, power Doppler ultrasound.

For the flexor carpi ulnaris tendinopathy, which presents without a tendon sheath, hypervascularity was assessed in the synovial proliferation for peritendinous and outside the synovial proliferation for extratendinous vessel assessment [22].

### Statistical methods

The statistical analysis was performed by using commercially available software (PASW Statistics 17; SPSS Inc., Chicago, IL, USA).

Interobserver agreement was tested with the Cohen kappa statistics and was interpreted according to the guidelines of Landis and Koch as poor, ≤0.20; fair, 0.21 to 0.40; moderate, 0.41 to 0.60; good, 0.61 to 0.80; or excellent, 0.81 to 1.00.

Differences between the CEUS and the PDUS groups regarding the severity scores were tested for significance by using the Wilcoxon test (in detail, differences regarding the detection of peri- and intratendinous vascularization, and the extent of detected vascularization).

Spearman rank correlation coefficients were used to assess a correlation between the different grading parameters (in detail, the correlation between detection of vascularization with PDUS and CEUS, respectively, and between extents of vascularity, peri-, and intratendinous vessel detection, tendinous vascularization, and enhancement of adjacent tissue and synovial thickening).

A value of *P *< 0.05 was considered significant for all tests.

## Results

Tenosynovial thickening was detected in all tendons examined (26 of 26; 100%). 40.4% (10 of 26 by observer 1, 11 of 26 by observer 2) were assessed with grade 1 (slight thickening of 2 to 4 mm), and 59.6% (16 of 26 and 15 of 26) showed sizable thickening of more than 4 mm (grade 2). A significant correlation between synovial thickening and extent of vascularity could not be found (*P *= 0.063 to 0.080; *r*_S _= 0.350 to 0.370). Excellent interobserver reliability could be achieved (κ = 0.920).

Tendinous vascularization was detected in 20 (69.2%) of 26 tendons with PDUS and in 26 of 26 tendons (100%) with CEUS.

The extent of peritendinous vascularization was assessed in relation to the axial plane of the whole synovial proliferation (see Table 2). With CEUS, a significantly (*P *< 0.001) greater amount of vascularized synovitis could be detected than by with PDUS. Interobserver agreement was calculated to be excellent with PDUS (κ = 0.937) and CEUS (κ = 0.920).

**Table 2:** Results of vascularity detection with PDUS and CEUS by using two different scoring systems

	Extent of vascularization		Peri-/intratendinous vessel detection	
		
	PDUS^a^	CEUS^a^	PDUS^b^	CEUS^b^
Grade 0	30.8% (8/26)	0.00% (0/26)	30.8% (8/26)	0.00% (0/26)

Grade 1	51.9% (14/26)^c^ (13/26)^d^	40.4% (10/26)^c^ (11/26)^d^	36.5% (10/26)^c^ (9/26)^d^	26.9% (6/26)^c^ (8/26)^d^

Grade 2	17.3% (4/26)^c^ (5/26)^d^	59.6% (16/26)^c^ (15/26)^d^	32.7% (8/26)^c^ (9/26)^d^	73.1% (20/26)^c^ (18/26)^d^

^a^CEUS more sensitive (higher grades) than PDUS with *P *< 0.001. ^b^CEUS more sensitive (higher grades) than PDUS with *P *= 0.001. ^c^Results of observer 1. ^d^Results of observer 2. CDUS, color Doppler ultrasound; PDUS, power Doppler ultrasound.

The comparison of the values regarding the detection of peri- and intratendinous vessels with PDUS and CEUS (see Table 2) showed that CEUS is significantly more sensitive in the detection of vascularization for both observers (*P *= 0.001). Interobserver reliability was calculated to be excellent by using both techniques (κ = 0.806 to 0.942).

No correlation between PDUS and CEUS regarding peri- and intratendinous vascularization was found (*r *= 0.25), whereas good to moderate correlation between PDUS and CEUS regarding the extent could be shown (*P *= 0.0009; *r *= 0.66).

Grading the intensity of tendinous vascularization by comparing tendinous enhancement with the enhancement in adjacent tissue showed the following results: grade 0, none; grade 1, 38.5%; and grade 2, 61.5%. Moderate correlation (*r*_S _= 0.51 to 0.60; *P *< 0.01) could be found between synovial thickness and the grade of tendinous in comparison with extratendinous enhancement. Perfect interobserver agreement could be achieved (κ = 1.00).

Overall, interobserver reliability was calculated to be excellent in every scoring (κ = 0.806 to 1.000; *P *< 0.001). None of the patients showed adverse reactions to the contrast agent.

## Discussion

PDUS has still not established itself as an imaging method in tendinopathy and enthesitis. D'Agostino *et al*. [23] suggested that this is due to the greater difficulty of assessing vascular blood flow with Doppler techniques of tendons in patients with spondyloarthropathies because of minor vessels compared with joint synovium.

By using CEUS, we probably overcome this problem because of the detection of vessels at the microvascular level. CEUS allows detection of low-volume blood flow in microvessels, which, by definition, is not possible, when using PDUS only. CEUS already was shown to be more sensitive than PDUS in the detection of intraarticular synovial vascularity and therefore better differentiation between active and inactive synovial thickening [15]. The use of the second-generation contrast agents improved sensitivity further.

Displaying microbubble enhancement in grey scale avoids Doppler-specific artifacts, maximizes contrast and spatial resolution, and enables the evaluation of the microcirculation (tissue perfusion) because of its independence of the speed of flow [15]. Computer-based quantification might, as quantitative analysis increases, discriminate validity (ability to detect change) of importance in clinical trials and should be further proven for therapeutic follow-ups in tendon diseases.

Because vascularization correlates with the destructive behavior of chronic inflammation, vessel imaging also is of pivotal importance in tendons. As new therapeutic strategies like biologics attack at different points in the signal cascade that induces angiogenesis as part of the immune reaction, a growing necessity for exact detection and quantification of vascularization at the angiogenic level might be of importance for therapy follow-up.

Moreover, our results concur with a multicenter study comparing PDUS with CEUS in joint examinations of RA patients [15]and with studies of Song *et al*. [16] and Schüller-Weidekamm *et al*. [24], which showed a significantly greater sensitivity of CEUS in detecting vascularity in joint synovium. We found that only peritendinous hypervascularity can be well depicted when using PDUS, whereas intratendinous vessels are depicted mainly when using CEUS; therefore, the correlation of PDUS and CEUS was good to moderate between both methods for peritendinous hypervascularity detection only (*P *= 0.0009; *r *= 0.66) and not for intratendinous vascularity detection. Good correlation but better sensitivity regarding CEUS and PDUS are in line with previously described vessel detection in joint synovitis. It can be speculated that, in more-advanced and aggressive disease, peritendinous synovitis invades the tendon, and CEUS enables earlier vessel detection in the tendon itself, reflecting progressive inflammation.

To our knowledge, this is the first study to compares CEUS and PDUS in the detection of vascularity in inflamed tendons. In the three studies of Adler *et al*. [25], Rudzki *et al*. [26], and Gamradt *et al*. [27], brightness-quantification software was used to calculate peak enhancement and rate of increase for assessing vascularity in the supraspinatus tendon and tendinosis. Studies that assess the reliability of tendon-vascularization scores are still rare [23,28,29], and the scoring systems used are widely variable.

Hence, because of lack of definitions for a scoring system of CEUS examinations in tendons, we had to establish a scoring system to grade tenosynovitis in terms of vascularity to compare the sensitivity of PDUS and CEUS. Our scoring system is based on vascularization distribution, taking into account intratendinous, peritendinous, and extratendinous vascularity, overall resulting in an excellent interobserver reliability (κ = 0.811 to 1.00). A more-refined assessment of vascularity in inflammatory rheumatic disease by using the unique potential of CEUS might be of importance for treatment follow-up, especially when therapies target the angiogenic level.

Morel *et al*. [17] offered some possible explanations for the failure to detect histologically obtained capillaries within tendons: a small distance between the vessels and the probe might cause too much pressure and therefore occlusion of the microvessels. Therefore, for best results, we used a gel-pad and avoided pressure.

The small diameter of the capillaries running through the tendon (<50 μm) is under the detection limit of PDUS, which might be a cause of contradictory results regarding the detection of vascularity in tendons. Different sensitivities of Doppler signal acquisition have been shown to have a great influence on US assessments, resulting in only moderate intermachine agreement [30,31], which might become a substantial problem for multicenter studies. As this study shows, by using CEUS, even slow flow in smaller vessels can be better detected when compared with PDUS in affected tendons.

To our knowledge, no published study detected vascularity in tendons of extensors and flexors of the wrist by using CEUS. According to the pathogenesis of tendon inflammation [7-10], we hypothesized that pathologic intratendinous vascularization is detectable solely in combination with peritendinous vascularization as a sign of invasive synovial proliferation, which might increase the risk for spontaneous tendon rupture [8]. This was the basis for the peri- and intratendinous vascularization score in our study, which therefore describes the progress of inflammation. In none of the tendons were intratendinous vessels observed without active peritendinous tenosynovial proliferation. However, we do not have a comparison of CEUS and PDUS in healthy tendons, but in previous studies, using CEUS, entheses are described as nonvascularized areas in healthy controls [17,32]. Furthermore, the peritendinous space within normal tendon sheaths is considered to be nonvascularized [33]. Nevertheless, further studies are required to assess normal tendons regarding potential intratendinous vascularity detectable with CEUS.

Milosavljevic *et al*. [29] measured tendon-sheath widening and graded it on a scale of 0 to 3: grade 0, tendon sheath diameter ≤0.3 mm; grade 1, diameter ≤2 mm; grade 2, diameter ≤4 mm; and grade 3, diameter >4 mm. Furthermore, they graded tendon and tendon-sheath tissue vascularity as follows: grade 0, no detectable PDUS signal; grade 1, mild vascularity (≤30% of synovial proliferations area); grade 2, moderate vascularity (≤60% of synovial proliferations area); and grade 3, severe vascularity (>60% of synovial proliferations area). With this scoring system, they achieved excellent inter- and intraobserver reliabilities (κ = 0.964 to 0.978). These gradings assure content validity (comprehensiveness) and can be used for PDUS as well as CEUS imaging. The extent of the inflamed area can be quantified (for example, as a parameter for follow-up examinations). Scoring peri- and intratendinous vascularization predetermined a three-grade scoring system. Therefore, we slightly modified the scoring system of Milosavljevic *et al*. [29] and obtained excellent interobserver reliabilities.

The comparison of tendinous and extratendinous enhancement describes the density of the capillaries in the inflamed area as a parameter of the inflammation intensity. Because capillary flow is not detectable in healthy adjacent tissue by using PDUS, only CEUS examination videos were graded by using this scoring. Further follow-up studies should focus on the clinical and prognostic value of this scoring.

Extensive tenosynovial invasion can complicate the assessment of altered tendons so that even a complete tendon rupture can become a diagnostic challenge, because tendon edema and inhomogeneous echo texture make difficult the evaluation of tendon continuity and tenosynovitis. Furthermore, inflammatory adhesions may cause limitations in the dynamic examination. Contrast-enhanced detection of vascularity may provide additional information for a better characterization of continuity and the amount of synovial proliferation.

Moreover, new therapeutic strategies like biologics attack at different points in the signal cascade that induces angiogenesis as part of the immune reaction. This leads to a further demand for sensitive detection and quantification of vascularization at the angiogenic level for therapy follow-up.

We must admit several limitations of the study: CEUS is considered to be costly and time consuming, although both factors are much less than those of contrast-enhanced MRI. Ultrasound contrast agents have some advantages over MRI contrast agents, because they are less likely to leak into the synovial fluid and to diffuse into the tissue; therefore, they can accurately demonstrate changes of the intravascular compartment.

Objective quantification of contrast enhancement seems promising for longitudinal assessment and comparison between studies. Standardization of measurements and interpretation of the characteristics of time/intensity curves suggest further investigation.

Furthermore, we did not include intraobserver reliability because the application of contrast media is already invasive when compared with PDUS, and is more intensive in cost and time required.

MRI would have been a nice gold standard, but because of the fact that MRI contrast agents diffuse into the extravascular compartment, it will not represent the true vascular compartment in hypervascularized synovium [34,35]. Therefore, PDUS was used as the standard reference method in this study. Song *et al*. [16] reported on a greater sensitivity of CEUS in the detection of vascularity in comparison to contrast-enhanced MRI in examining patients with knee osteoarthritis. They admitted that the objective quantification (calculated slope values) were not directly comparable.

Our sample size enabled us to identify significant findings and differences. Nevertheless, we believe that the significance of our data would have been greater with a larger cohort and additional observers to analyze the video sequences. Furthermore, comparing subjective and objective assessment by using brightness-quantification software might provide further information. We believe that computerized evaluation of intratendinous vascularization might be artefact prone because of slight changes in transducer tilt and the high baseline brightness of tendons itself that makes detection of faint enhancement insignificant.

## Conclusions

Our preliminary results show that CEUS is a promising tool to detect tendon vascularity with high sensitivity and excellent interobserver reliability when assessing intra- and peritendinous vascularity.

## Abbreviations

CCP: cyclic citrullinated peptide; CDUS: color Doppler ultrasound; CE-MRI: contrast-enhanced magnetic resonance imaging; CEUS: contrast-enhanced grey-scale ultrasound; ECU: extensor carpi ulnaris; ESR: erythrocyte sedimentation rate; FCR: flexor carpi radialis; MI: mechanical index; MRI: magnetic resonance imaging; OMERACT: outcome measures in rheumatology clinical trials; PDUS: power Doppler ultrasound; RA: rheumatoid arthritis; RF: rheumatoid factor; ROI: region of interest; SI: signal intensity; US: ultrasound.

## Competing interests

The authors declare that they have no competing interests.

## Authors' contributions

ASK designed the study, carried out the ultrasonographic examinations, helped to configure the scoring system, was one of the subjective observers, and helped to draft the manuscript and revised it critically. MF carried out the objective quantification, helped to configure the scoring system, was one of the subjective observers, and drafted and wrote the manuscript. RA, JG, MS, WJ, and MG participated in the design and coordination of the study and helped to draft the manuscript. GMF made substantial contributions to analysis and interpretation of data and performed the statistical analysis. All authors read and approved the final manuscript.
